# Smart healthcare engineering: transnational understanding of atmospheric corrosion processes in healthcare facilities

**DOI:** 10.3389/fpubh.2026.1822707

**Published:** 2026-07-20

**Authors:** Julia Claudia Mirza-Rosca, Ioan Aron

**Affiliations:** 1 Department of Materials Engineering and Welding, Transilvania University of Brasov, Brasov, Romania; 2 Department of Mechanical Engineering, University of Las Palmas de Gran Canaria, Las Palmas, Spain; 3 Department of Law, Transilvania University of Brasov, Brasov, Romania

**Keywords:** collaborative learning, corrosion, educational training, smart healthcare, transnational engineering

## Abstract

This work is a result of a pilot study integrated in an international project of collaboration and consists in the developing a comprehensive methodological framework to investigate the multifactorial nature of corrosion in hospitals across Spain, Romania and Turkey. By integrating multidisciplinary perspectives from engineering, materials science and law, the program prepares participants to address corrosion challenges through both technical competence and regulatory awareness. This pilot study conducted at Dr. Negrin Hospital (Spain) validated the methodology, involving 3 hospital professionals and 11 students with 3 academic staff from the University of Las Palmas de Gran Canaria. The study included environmental monitoring, laboratory experiments on electrochemical behavior and protective strategies against corrosion. Representative functional zones (including operating rooms, sterilization units, HVAC systems, intensive care units and patient wards) were instrumented with environmental monitoring devices to record pollutants, temperature, humidity and particulate matter. Metal coupons made of stainless steel, carbon steel, aluminum and zinc were exposed under simulated hospital conditions to assess corrosion rates. Data analysis combined quantitative and qualitative approaches to ensure methodological feasibility and reliability. The results emphasize that corrosion in healthcare settings has significant technical, economic and legal implications. Preventive strategies and continuous monitoring can reduce maintenance costs, enhance patient safety and ensure regulatory compliance. Ultimately, this work underscores the importance of multidisciplinary education and international collaboration in developing sustainable corrosion management practices for modern healthcare infrastructure.

## 1 Introduction

Smart healthcare represents a rapidly evolving field that integrates diverse engineering disciplines with advanced technological innovations designed for medical applications. As the demand for professionals skilled in smart healthcare engineering continues to increase, there is an imperative necessity to educate and train engineers with specialized competencies tailored to this field (1, 2). Consequently, in response to the requirements of the healthcare labor market, there exists a continuous necessity to revise and enhance engineering curricula, thereby preparing future generations of engineers capable of advancing intelligent and technology-driven healthcare systems.

Corrosion, the gradual degradation of materials (especially metals) due to chemical reactions with their environment, poses a significant risk in hospital settings. In healthcare facilities, corrosion can affect structural elements, medical devices, plumbing systems, HVAC units, sterilization equipment and electrical installations (3, 4). The consequences range from aesthetic deterioration and increased maintenance costs to impaired functionality, risk of contamination and patient safety hazards. Given that hospital environments combine aggressive agents as sterilant, disinfectants, etc. (5), variable atmospheric conditions (humidity, temperature and for exterior installations wind, dust, rain) and continuous equipment usage, understanding and controlling corrosion is of great importance for reliability, safety and cost efficiency.

In hospital maintenance engineering, a comprehensive understanding of corrosion processes is essential for ensuring the safety and longevity of critical infrastructure and medical equipment (6). An engineer specialized in hospital maintenance must therefore possess a strong foundation in the principles of corrosion science—encompassing electrochemical mechanism, environmental influences and material behavior under different conditions (7, 8). This knowledge enables the identification of early signs of material degradation and supports the selection of appropriate preventive strategies, including material selection, protective coatings, cathodic protection and environmental control. Furthermore, understanding the interaction between cleaning agents, disinfectants, humidity and temperature variations allows maintenance engineers to design effective maintenance protocols that minimize corrosion without compromising hygiene or safety standards (9–11).

Understanding corrosion processes contributes to the sustainability and resilience of healthcare facilities, providing a solid foundation for implementing next-generation E-health solutions and autonomous digital ecosystems powered by AI, IoT, and 5G connectivity (12–16).

Expertise in corrosion processes empowers hospital engineers to make informed decisions regarding maintenance planning, resource optimization and risk mitigation. By implementing evidence-based anti-corrosion practices, they contribute not only to extending the service life of hospital infrastructure but also to enhancing overall operational efficiency and sustainability within healthcare facilities.

**Figure 1:**
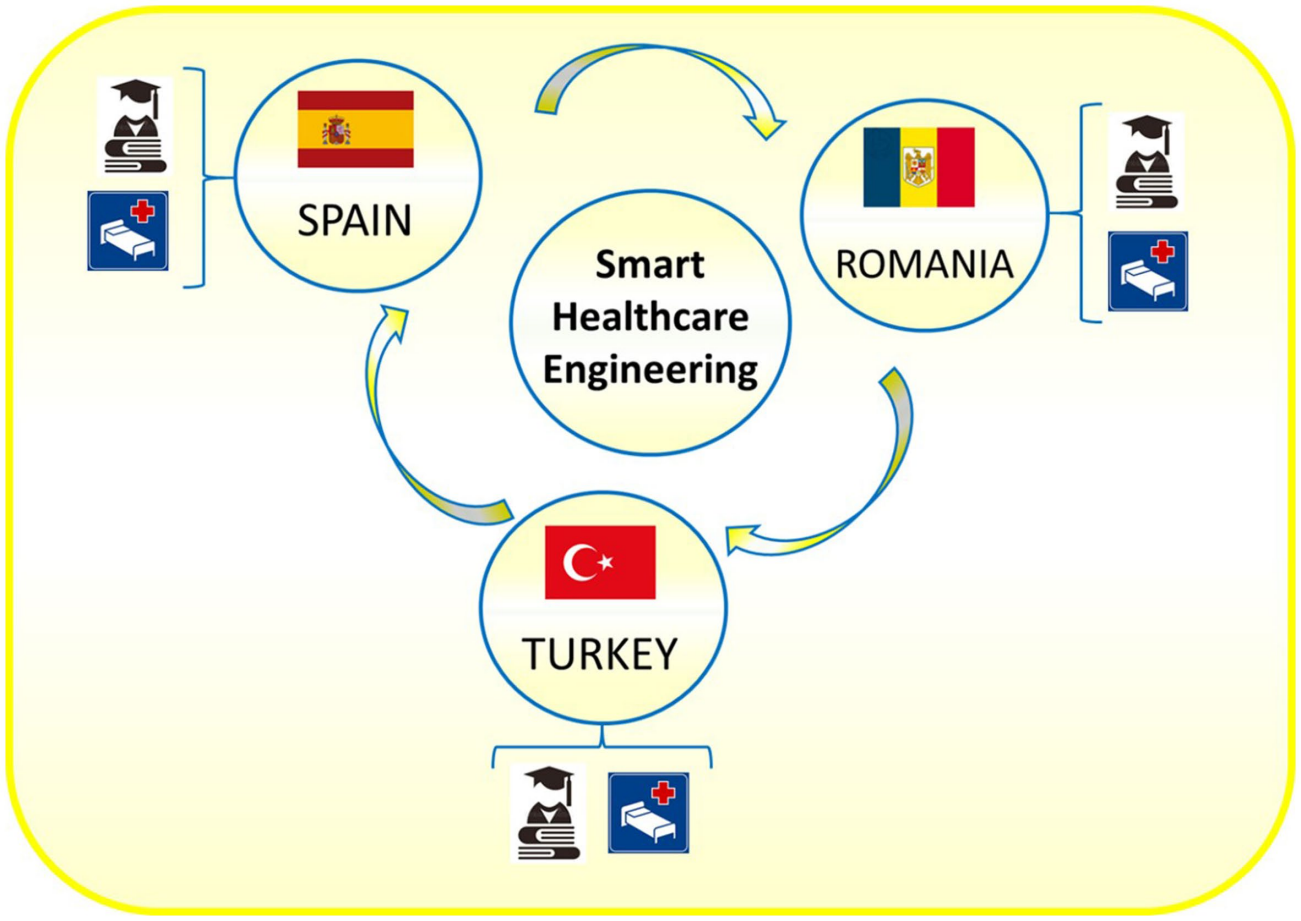
Map and logos illustrate the international partners involved in the Smart Healthcare Engineering project. The collaboration includes three universities and three hospitals from Spain, Romania and Turkey, highlighting the transnational scope of the educational and research program.

This study is presented as a pilot within a larger international project, Smart Healthcare Engineering (SHEng) (17), as a platform for training and collaboration. Figure 1 concretely illustrates the transnational aspect of our study, demonstrating who is involved and how the teams are distributed. A visual map of partners presents that this is not just a lab study, it is a coordinated effort involving universities (education and research) and hospitals (real world environments). The study aims to understand, monitor and reduce corrosion in healthcare settings through technical analysis, environmental measurements and the integration of legal and regulatory considerations, all within a framework of international staff participation.

This innovation report fosters the exchange of expertise and practical experience related to corrosion processes, prevention strategies and material performance in hospital environment. By transferring country-specific know-how and research outcomes across institutional, legal and cultural boundaries, the project enables participants to gain a broader and more integrated understanding of corrosion phenomena affecting healthcare infrastructure and prepares the students to navigate real-world professional contexts where technical expertise must align with legal accountability. Such international cooperation in education and applied research enriches the learning experience, promotes the dissemination of best practices and supports the development of a new generation of engineers equipped to address corrosion challenges in modern healthcare systems.

While existing research on corrosion in healthcare facilities has predominantly focused on either the isolated material performance in controlled laboratory conditions or the retrospective analysis of structural failures, there still remains a significant gap in the development of *in situ* monitoring protocols tailored to the multifactorial environments of moder hospitals. The novelty of the present work lies in its holistic and transnational approach, introducing a validated and comprehensive methodological framework that simultaneously integrates real time environmental monitoring within functional hospital zones with complementary electrochemical laboratory analysis. This dual approach allows for a direct and systematic correlation between specific atmospheric variables and the corrosion behavior of the hospital relevant materials. More than this, the study goes beyond the technical domain by embedding legal and policy perspective, framing corrosion not only as a maintenance issue but as a legal and safety responsibility. Pilot study spanning Spain, Romania and Turkey represents an unprecedent effort to standardize corrosion risk assessment across different regulatory, climatic and operational contexts, thus providing a transferable model for sustainable healthcare infrastructure management.

## 2 Project description

### 2.1 Procedure

In hospital settings, several airborne contaminants contribute to corrosion and one of the most important are disinfectants and sterilizing agents that vaporize or create aerosols as chlorine compounds, bleach, quaternary ammonium compounds, etc. (18, 19). Are also present volatile organic compounds (VOCs) from cleaning agents, paints, flooring materials, etc. (18).

For corrosive reactions the electrolyte is necessary to provide by humidity of the air and moisture from condensation, leaks and compressed air systems (20, 21).

Other studies (19, 20) reported that in contact with the exterior equipment there are gaseous pollutants such as sulfur dioxide (SO_2_), chloride (especially in coastal or poorly ventilated areas), nitrogen oxides, H_2_S, etc., which can form acids and attack protective oxide layers on materials.

Temperature fluctuations, rain and wind, dust and relative humidity are atmospheric variables that influence the time of wetness and the presence of contaminants, influencing the corrosion rates.

### 2.2 Objectives

1. One of the most important objectives of this study is to raise awareness among the students and hospital staff of the mechanisms and risk factors that facilitate corrosion in hospital environments.
2. Integrate legal and policy perspectives that are crucial for ensuring practices align with national and international standards, health and safety regulations and environmental protection laws.
3. Frames corrosion not only as a technical problem but also as a legal responsibility toward community well-being.
4. To understand the corrosion process mechanism.
5. To identify and measure atmospheric / environmental variables (humidity, temperature, pollutants concentrations, moisture levels) in hospital wards or service areas across Spain, Turkey and Romania.
6. To develop indicators that can quantify the severity or risk of corrosion in given hospital zones.
7. To test simple methodologies for monitoring corrosion risk in real hospital settings and compare across countries.
8. To suggest preventive or mitigate strategies based on findings.
9. Encourage understanding of risk allocation and liability clauses in technical service agreements related to facility maintenance.

### 2.3 Indicators

**Figure 2:**
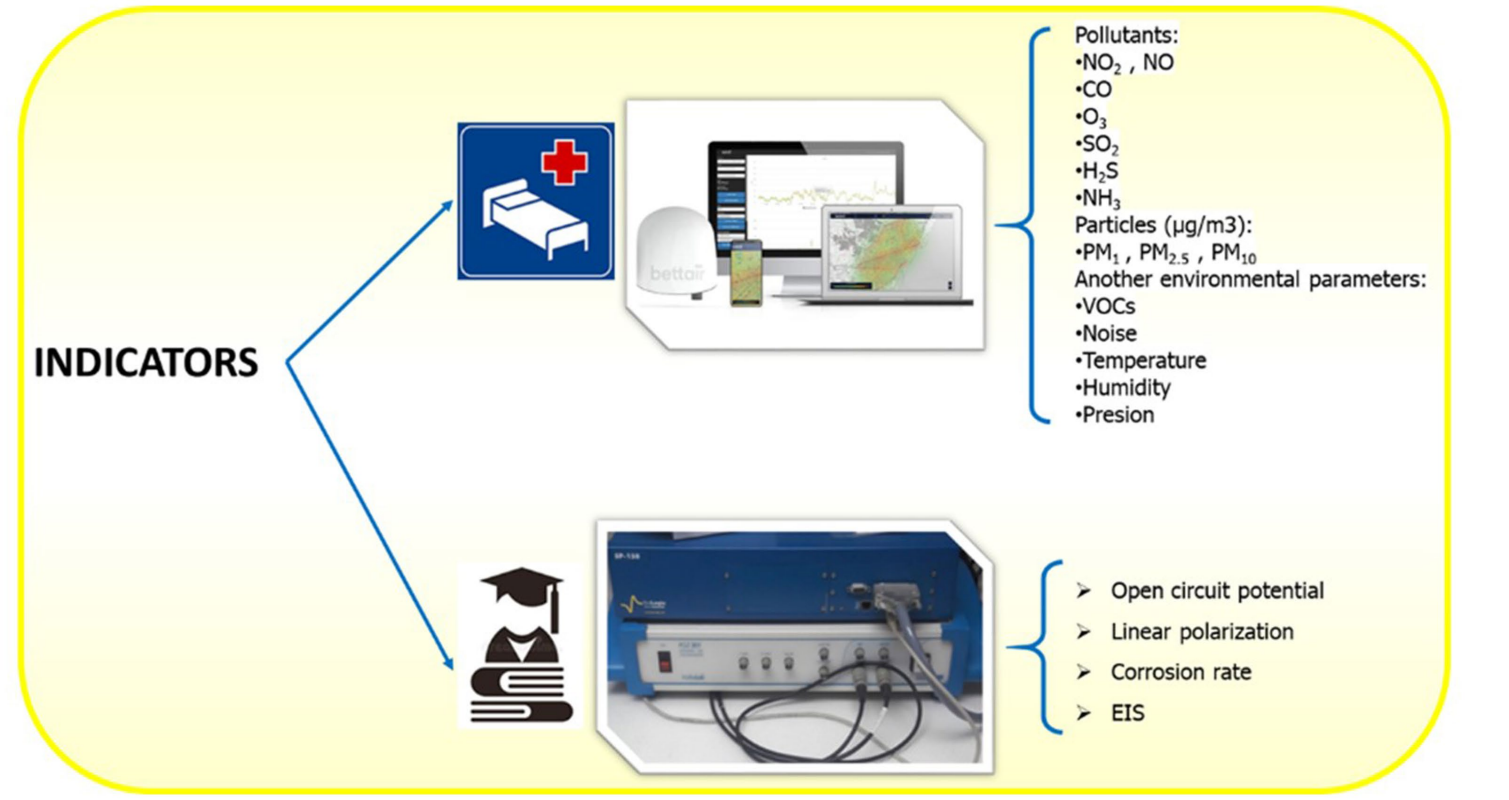
Schematic representation of the dual set of indicators used to monitor and quantify corrosion processes within the project. The indicators are categorized into environmental variables measured within the hospital zones (e.g., relative humidity, temperature, pollutant concentration) and electrochemical parameters measured within the university laboratory using exposed metal coupons.

The indicators are divided into two parts: one for the hospitals area and another for the university corrosion laboratory. In the hospital a Bettair device was installed, which is an advanced air quality monitoring system. This device can measure multiple air pollutants and environmental variables in real time, with cloud connectivity and high analytical accuracy. It is designed to monitor a wide range of pollutants (different gases and particulate matter) and environmental variables such as temperature, relative humidity, atmospheric pressure and ambient noise level (see Figure 2). The indicators for the hospital might include relative humidity (%), ambient temperature (°C), concentration of specific airborne pollutants (Cl^−^, SO_2_, VOCs). In the university a BioLogic SP-150 potentiostat was used (see Figure 2). It is a research-grade electrochemical instrument employed for corrosion studies, electrochemical kinetics and materials characterization. Its technical parameters are mainly related to voltage control, current measurement and impedance analysis. The indicators for the corrosion laboratory of the university consist in visual scoring, weight loss and electrochemical measurements such as open circuit potential, linear polarization, corrosion rate determination and electrochemical impedance spectroscopy of small coupons of common hospital-relevant alloys (stainless steel, aluminum, zinc, etc.) (see Figure 2).

### 2.4 Methodology

To systematically investigate the multifactorial nature of corrosion in healthcare environments, a comprehensive methodological framework was developed. The approach comprises five sequential phases, designed to ensure consistency, reproducibility and comparability of results across the participating hospitals and universities.

#### 2.4.1 Phase 1: selection of representative hospital zones

Representative functional areas were identified in each hospital to capture the range of environmental conditions influencing corrosion. These included critical zones such as operating rooms, sterilization units, heating, ventilation and air conditioning (HVAC) rooms, intensive care units (ICUs) and general patient wards. The selection aimed to encompass variations in temperature, humidity, pollutants and exposure to disinfectants and cleaning agents.

#### 2.4.2 Phase 2: installation of environmental monitoring instruments

Each selected zone was equipped with environmental monitoring instruments capable of recording parameters relevant to corrosion processes. These include sensors for pollutants, solid particles and atmospheric variables. Continuous data logging was implemented to ensure accurate characterization of the atmospheric conditions in each environment.

#### 2.4.3 Phase 3: employment of metal coupons

Small metal coupons composed of materials commonly used in hospital infrastructure—such as stainless steel, carbon steel, aluminum and zinc—were prepared and were analyzed in the laboratory simulating the environmental conditions of each representative zone from the hospital in order to determine the corrosion rate and the mechanism of corrosion process.

#### 2.4.4 Phase 4: data collection and analysis

Environmental data and corrosion parameters were systematically collected and daily recorded. The integration of these datasets enabled the identification of relationships between environmental variables and observed corrosion process.

#### 2.4.5 Phase 5: comparative evaluation across hospitals and countries

The final phase involved the comparative analysis of results obtained from the three participating countries—Spain, Turkey and Romania. Correlation analyses were conducted to determine which environmental factors and hospital zones exhibited the strongest association with corrosion intensity and morphology. This comparative assessment provided insight into how regional climatic conditions, maintenance practices and materials selection influence corrosion behavior in healthcare facilities.

### 2.5 Pilot study

**Figure 3:**
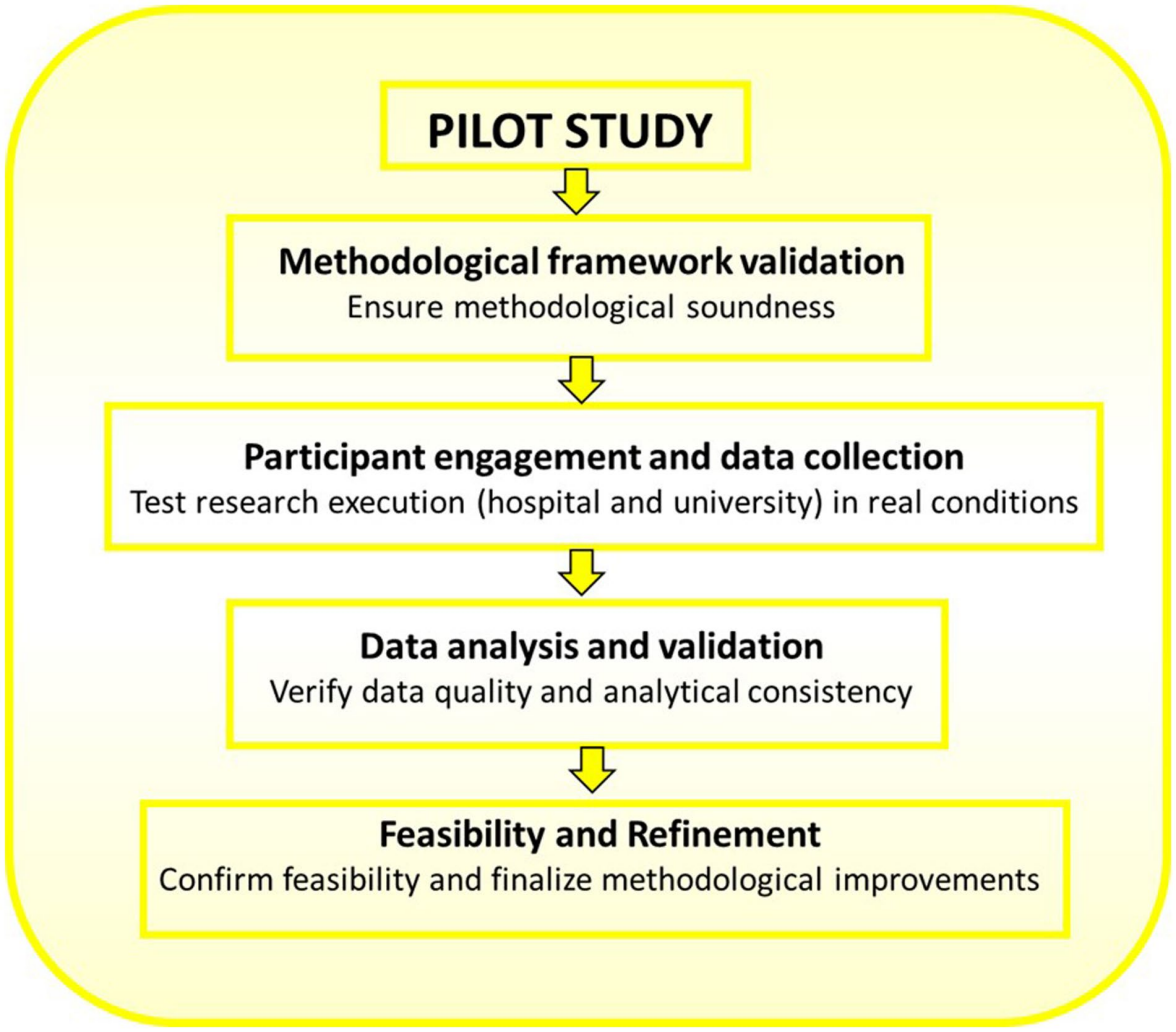
Schematic diagram depicting the structure and activities of the pilot study conducted at Dr. Negrin Hospital and the University of Las Palmas de Gran Canaria. The study is divided into hospital activities (installation of monitoring equipment and theoretical classes) and university activities (hand-on laboratory testing for electrochemical characterization).

The pilot study aims to validate different phases of methodology, identify potential limitations and confirm the overall feasibility of the research. It is structured into distinct phases. The workflow of the different tasks involved is presented in Figure 3. The pilot study involved participants from the University of Las Palmas de Gran Canaria, comprising 11 students and 3 academic staff members along with 3 professionals from partner hospital in the project, Dr. Negrin University Hospital in Spain. This composition ensured a representative balance between academic and professional expertise. The outcomes of the study were categorized into quantitative and qualitative data, allowing for a comprehensive analysis of both measurable parameters and experimental insights.

The metal coupons used for laboratory analysis were: AISI 304 and AISI 316, S235JR carbon steel, commercially pure Al (Al 1,100), commercially pure Cu (98.5%) and zinc Z1 grade. All samples were machined into 7.5 cm × 2.0 cm × 0.3 cm plates. Prior to each electrochemical test, the coupons were grounding with 1,200-grit emery paper and 0.1 μm colloidal alumina, degreases with acetone, rinsed with deionized water and dried. The exposed surface area for all tests was 1 cm^2^, with the rest of the coupon encapsulated in an epoxy resin.

For each simulated environmental condition (acid, basic and neutral), a minimum of three independent measurements were performed. Data acquisition for electrochemical impedance spectroscopy (EIS) was performed at open circuit potential over a frequency range of 100 kHz to 100 mHz with a 10 mV amplitude signal. Linear polarization resistance was measured by polarizing the sample ± 25 mV from the open circuit potential at a scan rate of 0.166 mV/s. Corrosion rates (mpy) were derived from the measured polarization resistance.

Data analysis was conducted using the Student’s t-test to determine the statistical significance of differences in corrosion rates between the three environmental conditions and the different materials. A *p*-value of less than 0.05 was considered statistically significant. The correlation coefficients between environmental parameters (e.g., temperature, relative humidity) and the measured corrosion rates were calculated using the Pearson correlation method.

**Figure 4:**
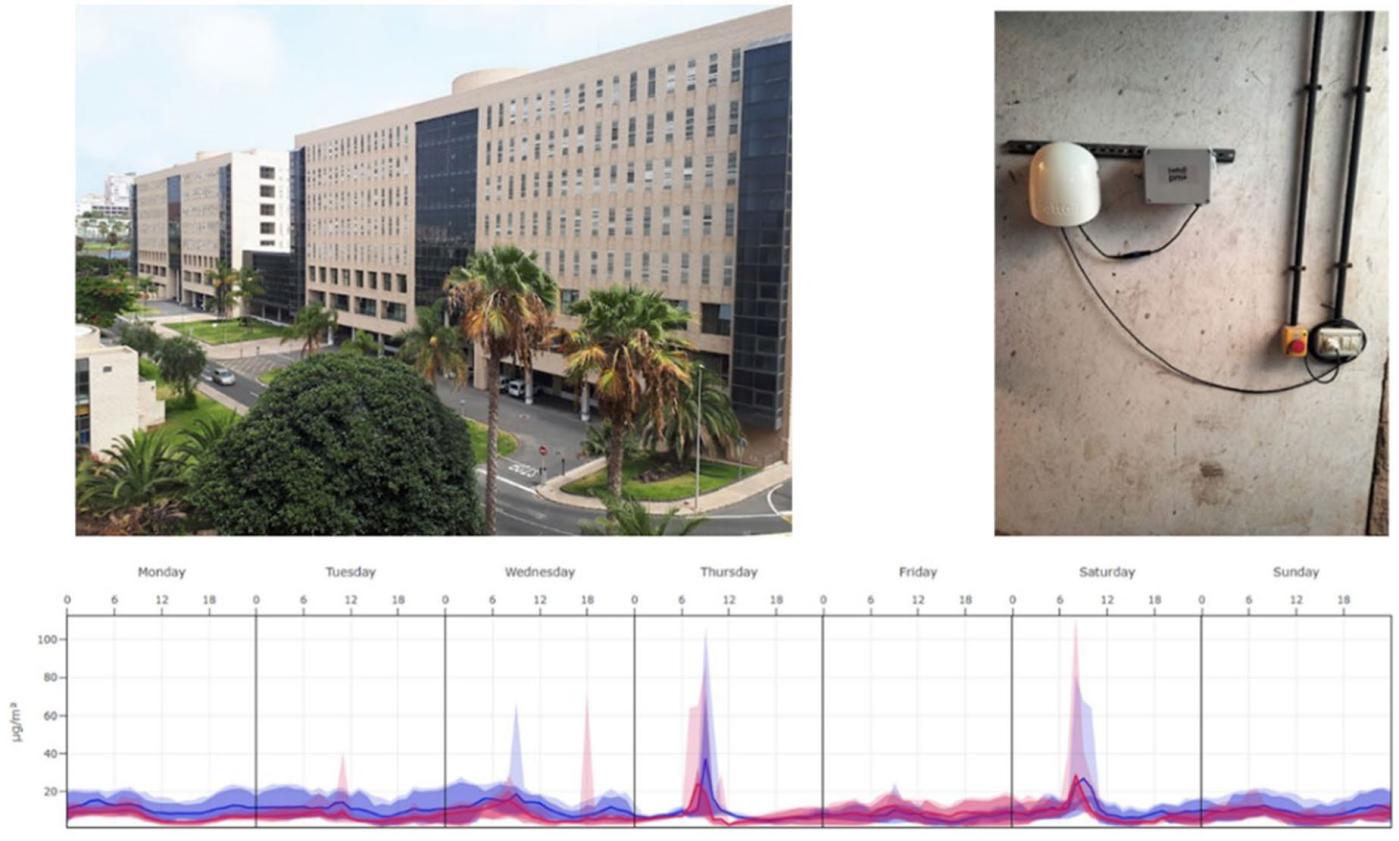
Photographs documenting the installation of the environmental monitoring equipment at Dr. Negrin hospital (left) and the data recording system (right). The equipment was strategically placed to capture representative atmospheric conditions in selected functional zones and data were continuously logged for subsequent correlation with corrosion analysis.

**Figure 5:**
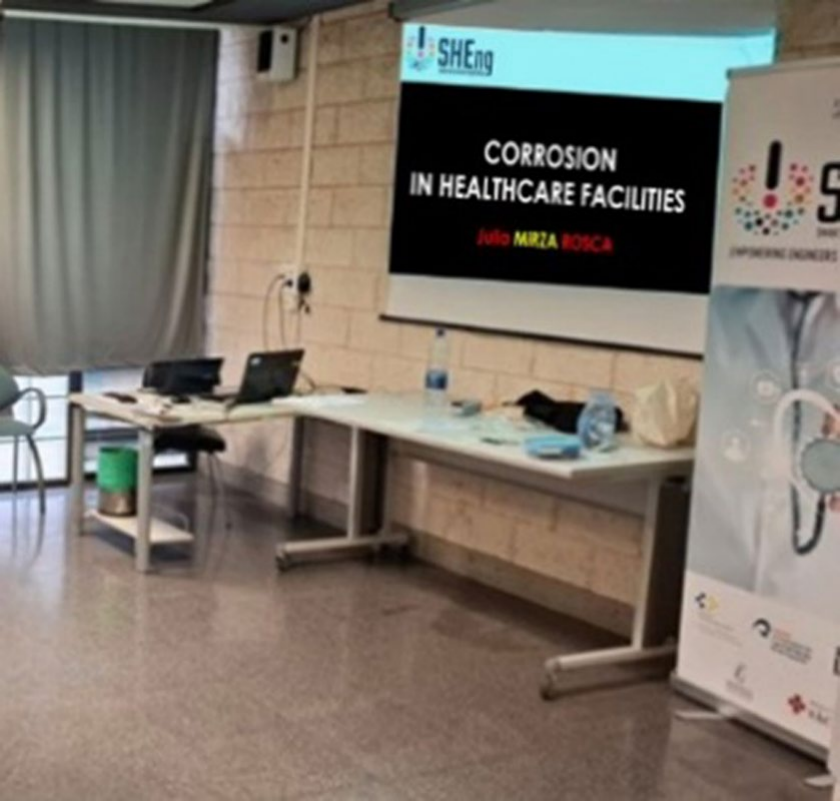
Photograph from the basic corrosion course (theoretical classes) conducted in a hospital classroom. The sessions aimed to familiarize participants with fundamental corrosion concepts, including definitions, classifications, protective methods and the influence of atmospheric variables and pollutants.

**Figure 6:**
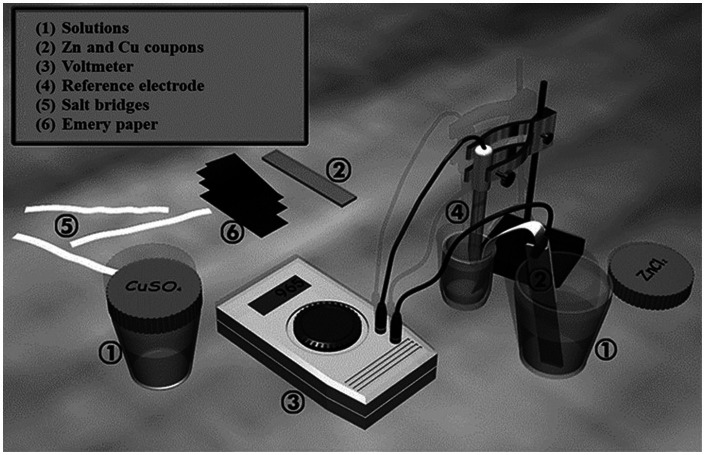
Photograph of a laboratory measurement setup used to determine the standard electrode potential of copper and zinc. The setup involves a multimeter connected to electrodes in an electrochemical cell, illustrating the practical methodology for measuring potential differences.

**Figure 7:**
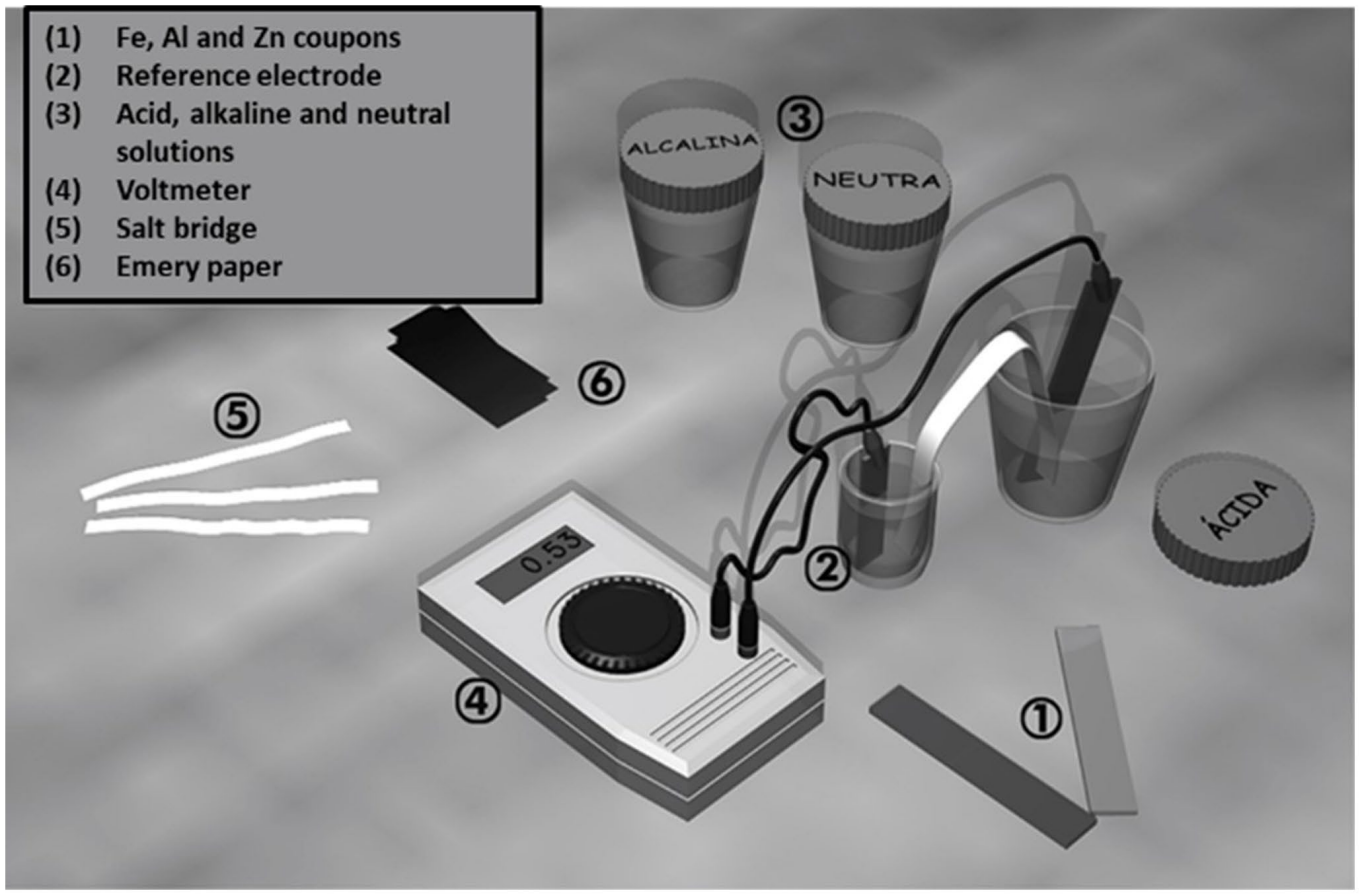
Experimental setup and graphical results (right) from the determination of corrosion potential for carbon steel, copper and zinc. Measurements were performed under simulated acidic, basic and neutral environments to assess the material specific response.

**Figure 8:**
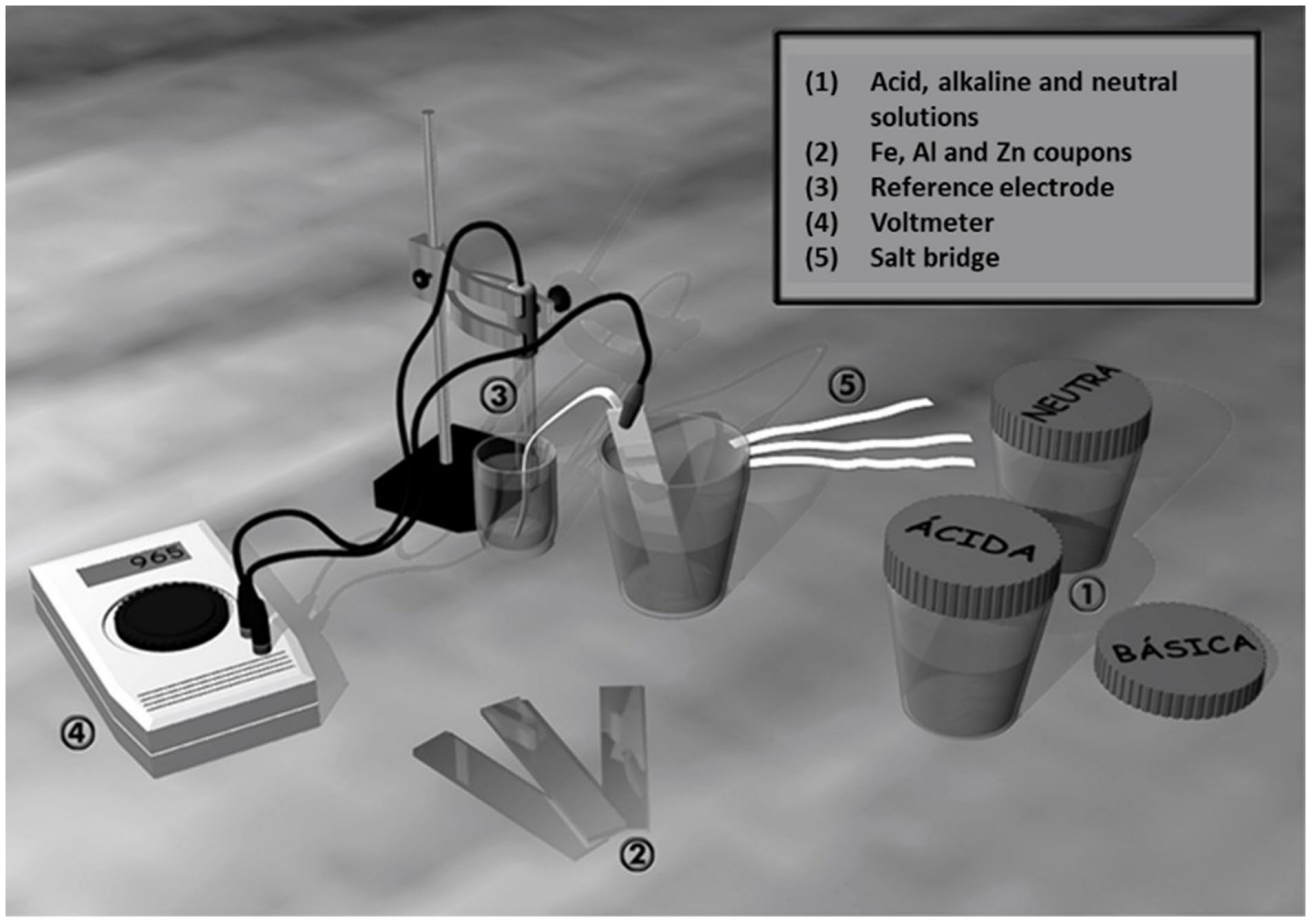
Photograph showing examples of sacrificial anodes used for cathodic protection against corrosion. The selection of an appropriate anode (e.g., magnesium, zinc) is a key learning objective of the laboratory exercises, demonstrating a practical mitigation strategy.

**Figure 9:**
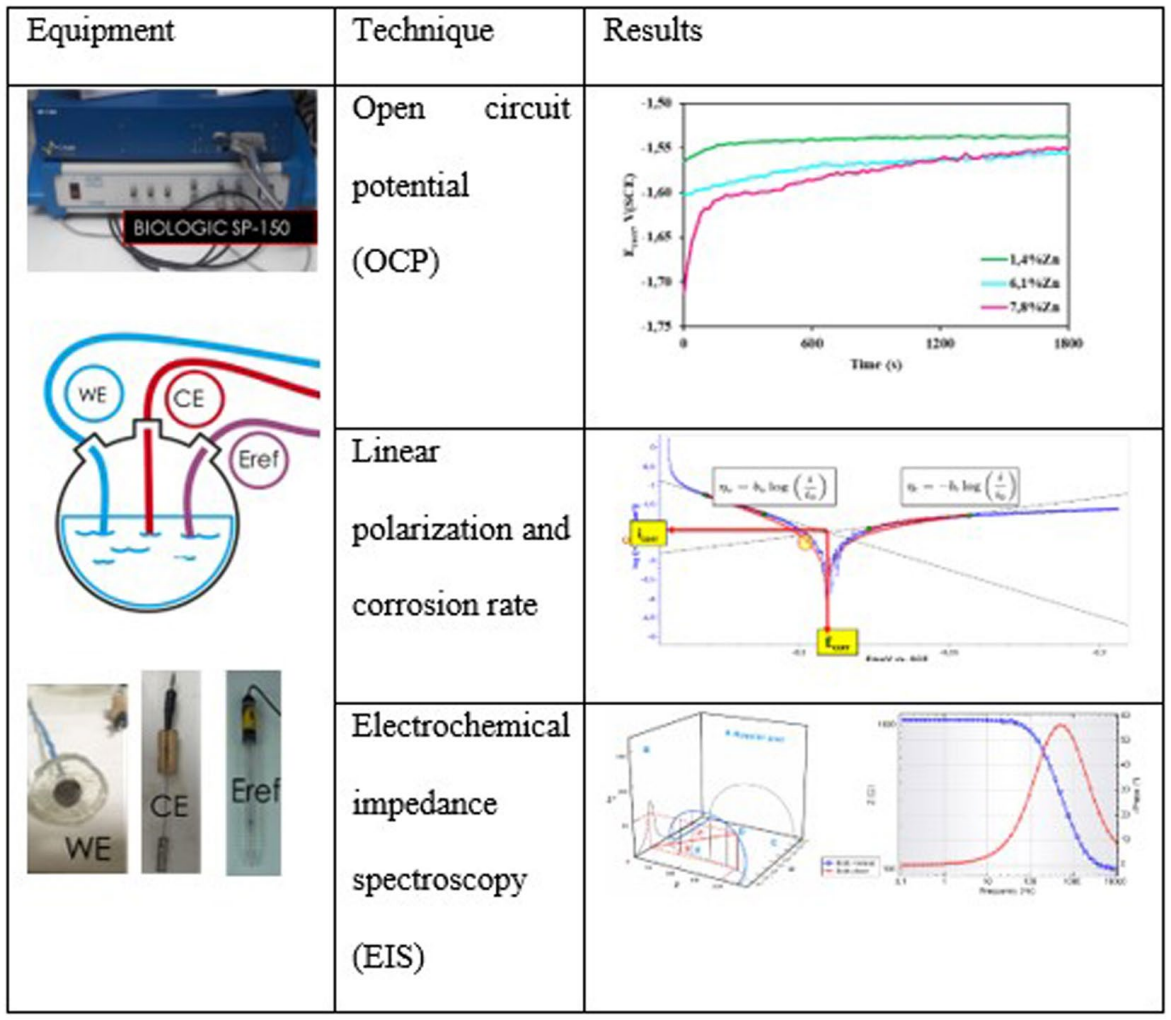
Graphical data outputs from advanced electrochemical measurements performed on selected materials. The plot shows results from Open Circuit Potential monitoring, linear polarization resistance for corrosion rate determination and Electrochemical Impedance Spectroscopy used to elucidate the underlying mechanism of the corrosion process (e.g., charge-transfer control).

The pilot study lasted 2 days, one in the hospital (the first 2 activities) and another in the corrosion laboratory (the rest of the activities) at the University of Las Palmas de Gran Canaria. The activities of the pilot study consist of:

- Installation in the hospital Dr. Negrin the equipment for the measuring of environmental parameters. In the hospital was installed the Bettair monitoring system (see Figure 4) and the data were daily recorded and compared with data provided by a public facility located a short distance from the hospital (22).
- Theoretical classes on basic corrosion concepts. The theoretical classes (see Figure 5) were carried out in the hospital classroom and consisted of two parts: one is the familiarization with fundamental concepts in corrosion: definition, classification, air pollutants, atmospheric variables, corrosion rate and methods of protection against corrosion and the second part with researches about the behavior of industrial materials in aggressive environments (23–25) and behavior of biomaterials in physiological environments (26).
- Determination in the laboratory of copper and zinc standard potential in laboratory conditions (see Figure 6) to assess the impact of accessories on the process: cable length, other additional resistances, laboratory temperature, etc.
- Determination of the corrosion potential for carbon steel, copper and zinc in the hospital conditions, choosing different pH (see Figure 7): solutions with chlorine compounds, bleach, quaternary ammonium compounds, cleaning agents, etc.
- Selection of a sacrifice anode for cathodic protection against corrosion (see Figure 8). The sacrificial anodes are used in engineering systems such as hot water tanks, plumbing networks, HVAC installations and underground pipelines to mitigate corrosion of metallic components exposed to aqueous environments.
- Electrochemical behavior in healthcare conditions. BioLogic SP-150 potentiostat was employed and different techniques were used to characterize the behavior of the analyzed industrial materials. Open circuit potential measurement was applied followed by Linear polarization and corrosion rate which determine if the materials remain protective in hospital environments and detect the risk of localized corrosion, which metals degrade faster and which zones (ICU, sterilization rooms, HVAC, etc.) are more aggressive. Electrochemical impedance spectroscopy analyzes metal/environment interface and assess protective coatings used in hospital infrastructure, detect early-stage corrosion before visible damage and evaluate how humidity and pollutants (from Bettair sensors) affect the corrosion mechanism (Figure 9).

## 3 Discussion

The pilot study provided essential insights into the feasibility and coherence of the proposed methodological framework and generated both quantitative and qualitative evidence supporting its practical application in hospital environments.

The installation of environmental monitoring equipment at Dr. Negrin Hospital functioned continuously for 7 days after setup, yielding reliable data on principal atmospheric parameters. Average temperature readings within the monitored zone ranged from 23.8 ± 0.7 °C, with relative humidity fluctuating between 47 and 65%, while particulate matter (PM2.5) concentrations averaged 6.7 μg/m^3^, slightly lower than the reference outdoor value of 7.1 μg/m^3^ provided by a nearby public monitoring station. These results confirmed the presence of distinct microclimatic variations within the hospital, especially in areas characterized by increased human activity and cleaning operations.

Laboratory investigations complemented these field observations. The open circuit potential (OCP) values for carbon steel, copper and zinc in neutral conditions were measured as −0.50 V, +0.34 V and −1.02 V respectively, versus the saturated calomel electrode (SCE). Linear polarization resistance (LPR) measurements yielded corrosion rates of approximately 0.14 mm/year for carbon steel, 0.04 mm/year for copper and 0.07 mm/year for zinc, highlighting the material-dependent behavior under identical test conditions. Electrochemical impedance spectroscopy (EIS) revealed that the corrosion process was primarily charge-transfer controlled, consistent with uniform corrosion mechanisms.

From an interpretative perspective, carbon steel exhibiting the highest corrosion rate, is therefore the most vulnerable material under the tested conditions, suggesting its limited suitability for use in hospital environments where humidity and chemical exposure are present. In contrast, copper showed the lowest corrosion rate and a more noble potential, indicating greater resistance and stability, making it a more appropriate candidate for certain applications. The intermediate behavior of zinc and its negative corrosion potential support its potential role a sacrificial material for cathodic protection. Furthermore, the identification of a charge transfer controlled mechanism implies that corrosion rates are strongly influenced by the electrochemical reactions at the metal/environment interface, which are affected by environmental parameters as temperature and pollutant concentration. This reinforces the importance of controlling indoor environmental conditions and of selecting appropriate materials to effectively mitigate corrosion in healthcare facilities. These quantitative results confirmed that the experimental setup and analytical techniques were adequate for capturing relevant corrosion dynamics in healthcare environments.

From a qualitative point of view, feedback collected from the 17 participants (11 students, 3 academic staff and 3 hospital staff) underscored the pedagogical and collaborative value of the exercise. Students reported that practical exposure to both field instrumentation and electrochemical testing significantly enhanced their comprehension of corrosion concepts, while hospital staff highlighted the relevance of the measurements for preventive maintenance planning. The interdisciplinary nature of the pilot study was repeatedly emphasized as a key strength, promoting as a key strength, promoting effective communication between academic and professional actors and fostering a shared understanding of corrosion management in medical facilities.

Nevertheless, the pilot study revealed several methodological weaknesses that must be addressed before full-scale implementation. The limited duration of the environmental monitoring (2 days of active observation and 1 week of passive recording) restricted the ability to detect temporal patterns such as diurnal humidity cycles or long-term pollutant accumulation. Furthermore, minor calibration drift in the humidity sensors was detected when cross-referenced with reference instruments, indicating the need for regular recalibration and sensor redundancy in the extended study. Finally, while the collaboration between academic and hospital teams was effective, logistical constraints within the hospital environment (for example the limited access to certain zones and strict sanitation schedules) posed challenges for the continuous acquisition of environmental data.

Complementing the quantitative findings, the qualitative feedback collected from the 17 participants provided critical insight into the practical and pedagogical value of the framework. A thematic analysis of the feedback was conducted and the consolidated perceptions are presented in Table 1. This feedback underscores that the transnational and collaborative nature of the project was perceived as its greatest strength, fostering a shared understanding of corrosion management that transcends disciplinary and cultural conditions.

**Table 1:** Qualitative feedback from the pilot study participants.

Theme	Representative feedback	% of respondents (*n* = 17)
Pedagogical value	“The hands-on experience with sensors and electrochemical methods made corrosion concepts far more tangible than theoretical lectures alone.”	88%
Relevance to practice	“This is directly applicable to our daily work. Understanding why we see certain types of failures helps us plan preventive measures more effectively.”	86%
Interdisciplinary strength	“Collaborating with engineers and seeing the legal implications was eye-opening: it’s not just selecting the right material, but also complying with safety standards.”	94%
Challenges encountered	“Navigating the hospital’s strict access and sanitation schedules was a major logistical hurdle for continuous monitoring.”	78%

## 4 Impact

**Figure 10:**
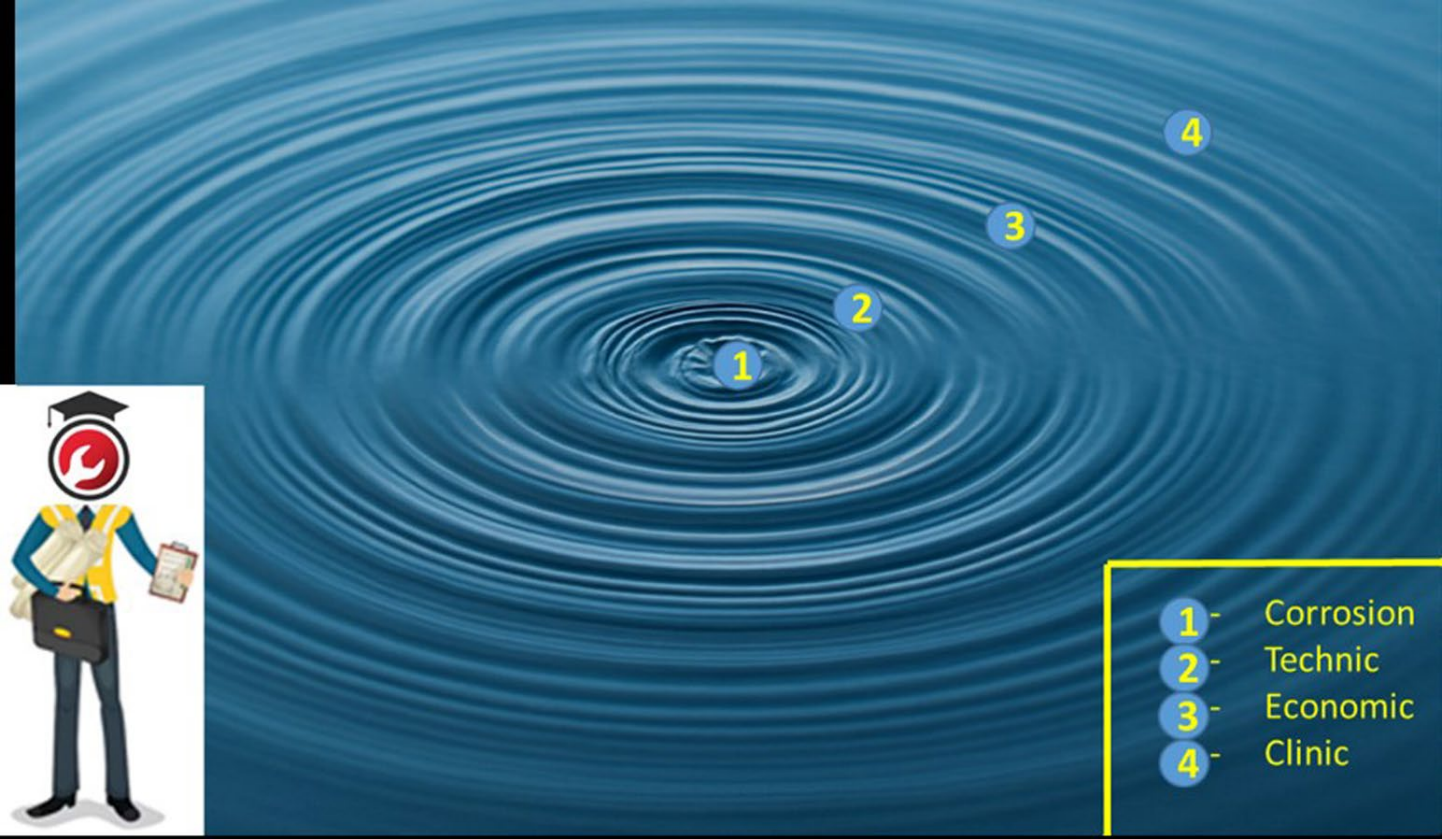
Diagram summarizing the multilevel impact of corrosion on healthcare facilities. The impacts are categorized into four levels: Technical (structural degradation, equipment failure, etc.), Clinical (microbial contamination, infection risk, etc.), Economic (increased maintenance, operational costs, etc.) and Safety/Legal (structural failure, non-compliance with regulations, etc.).

The findings from the pilot study, supported by evidence from scientific literature, highlight that corrosion represents a critical challenge in healthcare engineering, with impact that extend across technical, economical and clinical domains. As illustrated in Figure 10, the impact of corrosion can be understood as a ripple effect, where the initial material degradation (1—corrosion) progressively propagates through technical (2), economical (3), and clinical (4) levels, amplifying its consequences across the entire healthcare effects, affecting infrastructure performance, operational costs and ultimately patient safety. Corrosion affects not only the longevity and reliability of hospital infrastructure and medical devices but also the safety and quality of patient care. Its multifactorial nature requires comprehensive understanding and proactive management strategies to minimize both direct and indirect consequences.

Corrosion accelerates the deterioration of metal components within essential hospital systems such as sterilization units, HVAC systems and water distribution networks. Components exposed to high humidity, disinfectants or saline environments (such as those in operating theatres or intensive care units) are particularly vulnerable. This degradation results in more frequent maintenance interventions, premature equipment replacement and increased operational expenses. The loss of efficiency in corroded systems, such as reduced heat transfer or impaired fluid flow, further compound these costs.

Corroded surfaces in robotic systems, sensors and metallic interfaces present a significant challenge to hospital hygiene and infection prevention protocols (27, 28). The formation of pits, crevices and irregular textures on metallic surfaces creates ideal environments for microbial colonization and biofilm formation. These microenvironments are resistant to standard sterilization procedures, posing potential risks of cross-contamination and nosocomial infections. The economic impact of corrosion extends beyond the immediate costs of repair and component replacement. Indirect costs, including system downtime, patient relocation, infection control measures and potential regulatory penalties for non-compliance with safety standards, can be substantial. Hospitals operating under constrained budgets may face significant financial strain, underscoring the importance of implementing preventive maintenance strategies and corrosion monitoring programs to ensure sustainable facility management.

The findings of the study not only confirm the presence and impact of corrosion in healthcare environments but also indicate clear pathways for mitigate it. Based on the measured environmental conditions and electrochemical analyses, several practical actions can be implemented. First, stricter control of indoor environmental conditions, particularly relative humidity and air quality, should be achieved through optimized HVAC operation and air filtration systems. Second, the selection of materials must be adapted to the aggressiveness of specific hospital zones, prioritizing corrosion resistant alloys and/or protective coatings over more vulnerable materials such as carbon steel. Third, the application of preventive techniques such as use of sacrificial anodes can significantly reduce the corrosion in critical systems. In addition, the integration of continuous environmental and electrochemical monitoring would enable early detection of corrosion processes and support predictive maintenance strategies. Finally, improvements in maintenance protocols, including the careful selection of cleaning agents and the scheduling of inspections based on environmental risk factors are essential to minimizing the damage related to corrosion. Together, these measures provide a practical framework for translating experimental findings into effective corrosion management in healthcare facilities.

## 5 Conclusion

The analysis of atmospheric corrosion impacts within healthcare engineering underscores the necessity of adopting an integrated approach that combines preventive maintenance, material science and engineering management. The hospital environment presents unique challenges to material durability due to the coexistence of high humidity, temperature fluctuations, disinfectant exposure and the continuous operation of complex systems. These factors accelerate corrosion processes and demand the implementation of structured maintenance programs rooted in scientific understanding and long-term planning. Consequently, corrosion management must become a fundamental component in hospital engineering strategies rather than isolated corrective practice.

A comprehensive corrosion management framework should encompass regular environmental monitoring, risk-based inspection schedules and systematic data recording on corrosion-prone zones and materials. Predictive maintenance tools (such as sensor-based monitoring and data analytics) can assist engineers in anticipating failures before they occur, thus optimizing intervention timing and reducing maintenance costs. Additionally, integrating corrosion prevention principles into the design and material selection stages of hospital infrastructure can yield significant benefits. For example, the use of corrosion resistant materials, protective coatings and controlled ventilation systems can substantially reduce corrosion rates in critical environments such as operating theatres and sterilization units.

Beyond technical measures, the educational dimension plays an equally important role. Maintenance engineers must be equipped with specialized training in corrosion science, encompassing electrochemical mechanisms, materials performance and protective technologies. Projects such as the Smart Healthcare Engineering (SHEng) initiative demonstrate the value of international academic-industrial collaboration in developing these competencies. By fostering the exchange of knowledge among the universities and hospitals across different climatic and cultural contexts, such programs enable the transfer of effective corrosion mitigation strategies and the harmonization of maintenance standards.

From a managerial perspective, embedding corrosion awareness within hospital maintenance policies promotes a culture of prevention and accountability. Decision-makers should prioritize long-term asset management over short-term reductions, recognizing that investments in corrosion monitoring and prevention yield measurable returns in operational reliability, safety and regulatory compliance. Furthermore, the adoption of standardized corrosion assessment protocols can facilitate benchmarking across healthcare institutions, contributing to continuous improvement and best practice dissemination at both national and international levels.

So, the mitigation of corrosion in healthcare facilities is not merely a technical challenge but a multidisciplinary endeavor that integrates engineering science, management, law and education. Addressing it proactively ensures not only the structural and operational integrity of hospital systems but also the continuity of safe and high-quality patient care. By institutionalizing corrosion knowledge and prevention practices, healthcare engineering can evolve towards a more sustainable, efficient and resilient model of infrastructure management.

## Data Availability

The raw data supporting the conclusions of this article will be made available by the authors, without undue reservation.
